# Apolipoprotein E gene polymorphism and the risk of intracerebral hemorrhage: a meta-analysis of epidemiologic studies

**DOI:** 10.1186/1476-511X-13-47

**Published:** 2014-03-12

**Authors:** Rongjun Zhang, Xiaofeng Wang, Zongchun Tang, Jianxin Liu, Shuzhen Yang, Youbing Zhang, Yijun Wei, Wenyin Luo, Jun Wang, Jialong Li, Bobo Chen, Kunhu Zhang

**Affiliations:** 1 Department of Neurosurgery, Third Hospital of Chinese PLA, 45 Dongfeng Road, Jin Tai District, Baoji 721004, Shanxi Province, China

**Keywords:** Apolipoprotein E, Intracerebral hemorrhage, Gene polymorphism, Meta-analysis

## Abstract

**Background:**

Studies investigating the association between the apolipoprotein E (APOE) gene polymorphism and the risk of intracerebral hemorrhage (ICH) have reported conflicting results. We here performed a meta-analysis based on the evidence currently available from the literature to make a more precise estimation of this relationship.

**Methods:**

Published literature from the National Library of Medline and Embase databases were retrieved. Odds ratio (OR) and 95% confidence interval (CI) were calculated in fixed- or random-effects models when appropriate. Subgroup analyses were performed by race.

**Results:**

This meta-analysis included 11 case–control studies, which included 1,238 ICH cases and 3,575 controls. The combined results based on all studies showed that ICH cases had a significantly higher frequency of APOE ϵ4 allele (OR= 1.42, 95% CI= 1.21,1.67, P<0.001). In the subgroup analysis by race, we also found that ICH cases had a significantly higher frequency of APOE ϵ4 allele in Asians (OR= 1.52, 95% CI= 1.20,1.93, P<0.001) and in Caucasians (OR= 1.34, 95% CI= 1.07,1.66, P=0.009). There was no significant relationship between APOE ϵ2 allele and the risk of ICH.

**Conclusion:**

Our meta-analysis suggested that APOE ϵ4 allele was associated with a higher risk of ICH.

## Introduction

Intracerebral hemorrhage (ICH) occurs at an annual incidence rate of 15 to 19 per 100,000 [1]. ICH accounts for approximately 15% of acute strokes in the United States and 22–35% in Asian populations [2-5]. ICH can be a devastating type of stroke, and the 30-day case mortality rate of ICH is 40% to 50%. [2,6]. A pooled prospective study found that the risk factors for ICH were older age, African-American ethnicity, hypertension, lower LDL-C, and lower triglycerides [7]. ICH has been shown to have important genetic and environmental risk factors.

Apolipoprotein E (APOE) gene, located on the long arm of chromosome 19, codes for a 299-amino acid protein (apoE). ApoE is a polymorphic glycoprotein involved in cholesterol transport and cell membrane maintenance and repair [8,9]. *APOE* has three common alleles: epsilon 2 (ϵ2), ϵ3, and ϵ4 that encode the three major isoforms of apoE: E2, E3, and E4, which performs isoform-dependent neurotrophic and antioxidant functions [8,10-12]. Each person has 2 alleles that together compose that person’s *APOE* genotype (e.g., ϵ2/ϵ3 or ϵ3/ϵ3). *APOE* is one of the most widely studied genes in vascular and neurodegenerative diseases [13].

Recently, some studies have been conducted to clarify the association between *APOE* gene polymorphisms and the risk of ICH [14-24]. However, previous studies investigating the association have reported conflicting results [25,26]. We here performed a meta-analysis based on the evidence currently available from the literature to make a more precise estimation of this relationship.

## Materials and methods

### Literature search strategy

We used a detailed electronic search strategy in Medline and Embase from 1950 to the end of March 2013. Two authors independently searched the databases using following key words in all relevant combinations: ‘cerebral’ or ‘intracerebral’ or ‘intracranial’, ‘hemorrhage’, ‘apolipoprotein E*’ or ‘ApoE*’, ‘polymorphism’ or ‘allele’ or ‘genotype’ or ‘variant’. The search was conducted without limitation on language. The reference lists of all retrieved publications were scrutinized for additional studies. If studies had partially overlapping subjects, the smaller dataset was excluded. If necessary, we attempted to contact the principal investigators of retrieved articles to require additional data.

### Inclusion and exclusion criteria

The following criteria were used to include published studies: (i) independent epidemiological studies (for humans only); (ii) a clear description of *APOE* allele in ICH cases and controls; (iii) sufficient allele data were presented to calculate the odds ratio (OR) and 95% confidence interval (CI); Major reasons for exclusion of studies were (i) no control; (ii) not an original paper (e.g. review or letter etc.); (iii) duplicate publications.

### Data extraction

Data were extracted by two authors independently, and disagreements were resolved by consensus. When a study did not explicitly report one or more of the requested data, we contacted the author of the study for additional details. The following data were extracted: the last name of the first author, publication year, country, study design, genotyping method, sample size and the results of studies.

### Statistical analysis

All analyses were performed using STATA 11.0 (Stata-Corp LP, College Station, TX, USA). The Mantel-Haenszel method for fixed effects and the Der-Simonian-Laird method for random effects were used to estimate pooled OR and corresponding 95% CI. Meta-analysis heterogeneity was quantified by computing Cochrane’s Q and corresponding *P*-value and I^2^ (percent of effect size attributable to heterogeneity). We used fixed-effects methods if the result of the Q test was not significant. Otherwise, we calculated pooled estimates and confidence intervals assuming a random-effects model. Also, subgroup analyses were performed on the basis of race. In this study, *P* < 0.05 was considered statistically significant. Publication bias was assessed by visual inspection of funnel plots, the Begg's rank correlation method and the Egger's weighted regression method.

## Results

### Study characteristics

Characteristics of studies included in the meta-analysis are summarized in Table 1. Our initial search identified 108 studies according to the search words. Through the step of screening the title, abstracts, 85 articles were excluded, leaving 23 articles for full publication review. Of these, 12 were excluded [25-36]. Finally, a total of 11 studies were included in our meta-analysis [14-24], which included 1,238 ICH cases and 3,575 controls. Of those, three studies were population-based case–control studies, and eight studies were hospital-based case–control studies. Studies were conducted in USA, United Kingdom, Portugal, Japan, India and China. The frequencies of apolipoprotein E alleles of studies included in the meta-analysis were shown in Table 2.

**Table 1:** Characteristics of studies included in the meta-analysis

**Study (author, year)**	**Design**	**Study period**	**Population (country)**	**Genotyping method**	**No. of cases**	**No. of controls**
Nakata 1997	HCC	1992-1995	Asians (Japan)	PCR	38	38
McCarron 1998	HCC	DNR	Caucasians (United Kingdom)	PCR	71	406
Garcia 1999	PCC	DNR	Caucasians (Portugal)	PCR-RFLP	48	173
Kokubo 2000	PCC	1997-1999	Asians (Japan)	PCR-RFLP	84	1126
Catto 2000	HCC	1997	Caucasians (United Kingdom)	PCR	60	289
Chowdhury 2001	HCC	1998-1999	Asians (Japan)	PCR-RFLP	80	190
Woo 2002	PCC	1997-2000	Caucasians (USA)	PCR	188	366
Woo 2005	HCC	1997-2002	Caucasians (USA)	TaqMan	172	339
Chen 2009	HCC	DNR	Asians (China)	PCR	217	280
Zhang 2012	HCC	2008-2010	Asians (China)	PCR-RFLP	180	180
Misra 2012	HCC	DNR	Asians (India)	PCR	100	188

*Abbreviations*: *HCC* Hospital-based case–control, *PCC* Population-based case–control, *DNR* Data not reported, *PCR* Polymerase chain reaction, *RFLP* Restriction fragment length polymorphism.

**Table 2:** Frequencies of apolipoprotein E alleles of studies included in the meta-analysis

**Study (author, year)**	**ϵ2 of cases**	**ϵ3 of cases**	**ϵ4 of cases**	**ϵ2 of controls**	**ϵ3 of controls**	**ϵ4 of controls**
Nakata 1997	3	32	3	2	32	4
McCarron 1998	18	89	35	66	599	147
Garcia 1999	4	84	8	14	298	34
Kokubo 2000	14	131	23	103	1913	236
Catto 2000	5	95	20	44	446	88
Chowdhury 2001	2	146	12	13	333	34
Woo 2002	39	96	53	64	206	96
Woo 2005	15	118	39	25	266	48
Chen 2009	33	359	42	44	479	37
Zhang 2012	26	280	54	26	314	20
Misra 2012	7	183	10	15	343	18

### Quantitative synthesis

**Figure 1:**
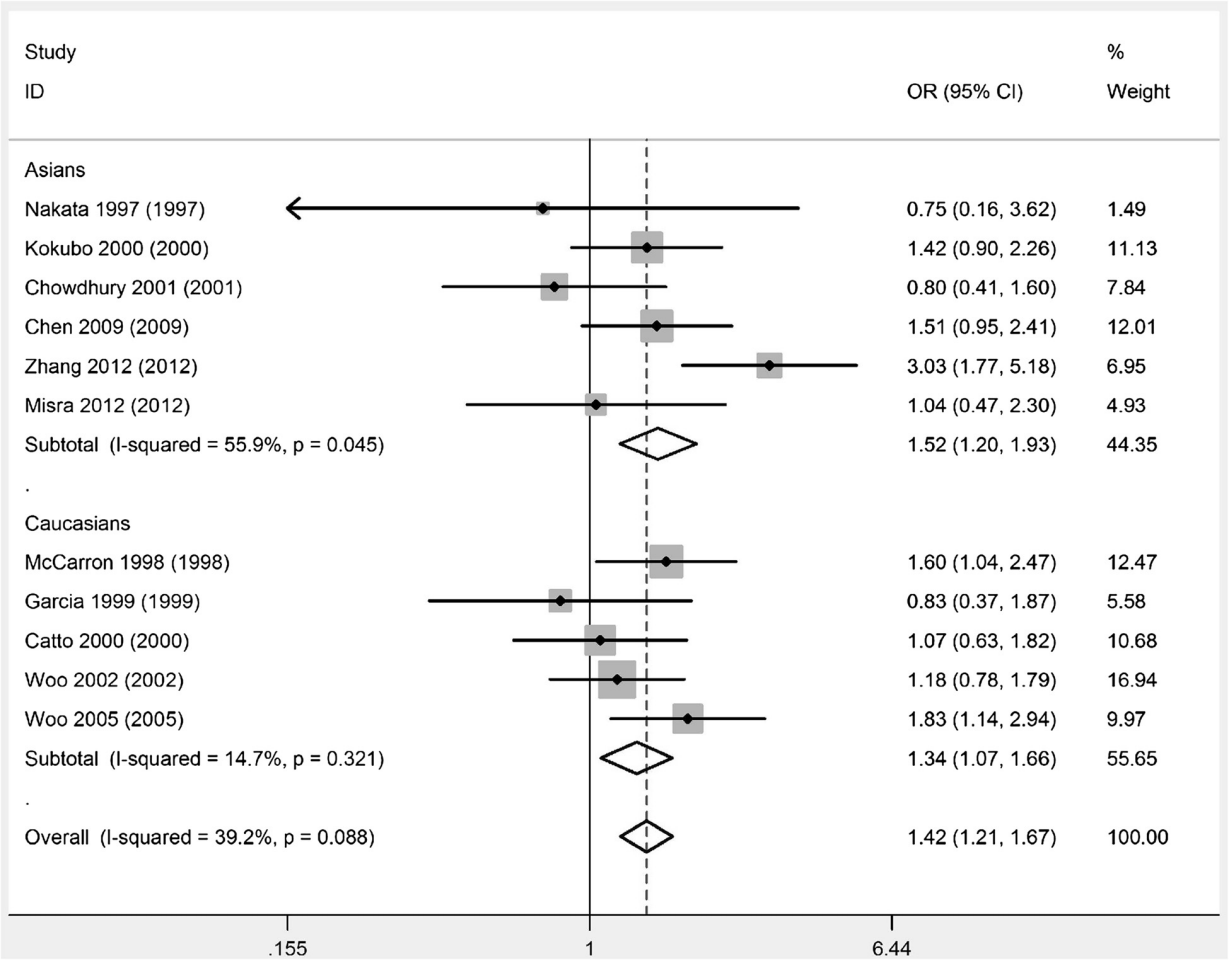
**Meta-analysis of *APOE *** **alleles and ICH (ϵ4 versus ϵ3).**

**Figure 2:**
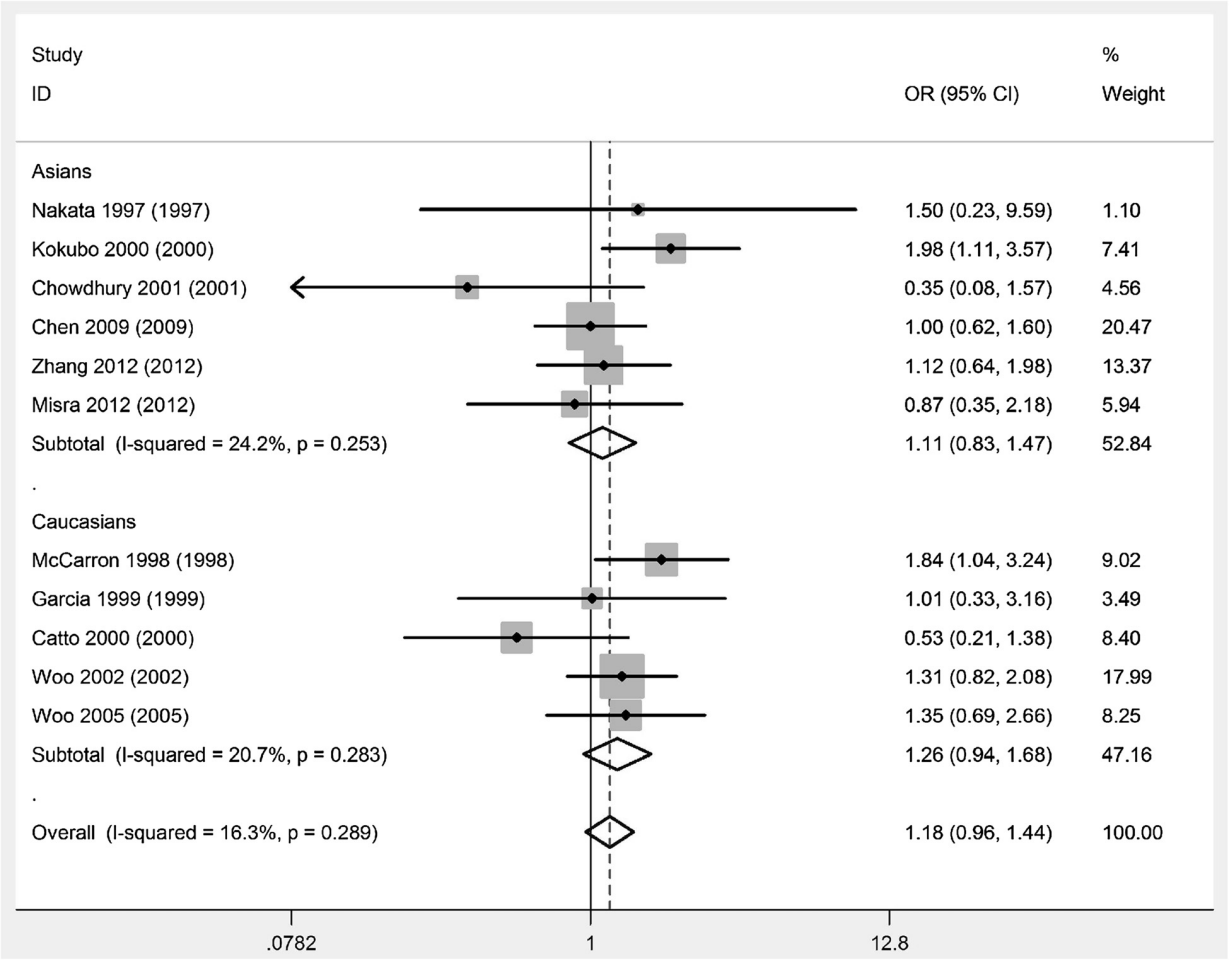
**Meta-analysis of *APOE *** **alleles and ICH (ϵ2 versus ϵ3).**

The combined results based on all studies showed that ICH cases had a significantly higher frequency of *APOE* ϵ4 allele (OR= 1.42, 95% CI= 1.21,1.67, *P*<0.001) (Figure 1) (Table 3). In the subgroup analysis by race, we also found that ICH cases had a significantly higher frequency of *APOE* ϵ4 allele in Asians (OR= 1.52, 95% CI= 1.20,1.93, *P*<0.001) and in Caucasians (OR= 1.34, 95% CI= 1.07,1.66, *P*=0.009) (Figure 1) (Table 3). There was no significant relationship between *APOE* ϵ2 allele and the risk of ICH (Figure 2) (Table 3).

**Table 3:** Meta-analysis of apolipoprotein E alleles and intracerebral hemorrhage risk

	**No. of studies**	**OR (95% CI)**	***P *** **of OR**	***P *** **of heterogeneity**	**OR (95% CI)**	***P *** **of OR**	***P *** **of heterogeneity**
		**ϵ4 versus ϵ3**			**ϵ2 versus ϵ3**		
All	11	1.42 (1.21,1.67)	<0.001	0.09	1.18 (0.96,1.44)	0.11	0.29
Asians	6	1.52 (1.20,1.93)	<0.001	0.05	1.11 (0.83,1.47)	0.47	0.25
Caucasians	5	1.34 (1.07,1.66)	0.009	0.32	1.26 (0.94,1.68)	0.12	0.28

*Abbreviations*: *OR* Odds ratio, *CI* Confidence interval.

### Heterogeneity analysis and publication bias

**Figure 3:**
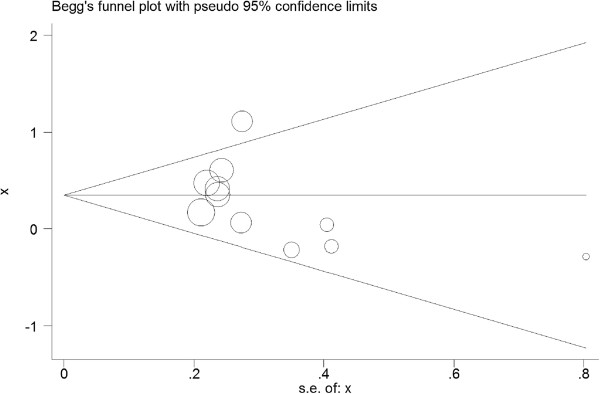
**Begg’s publication bias plot of *APOE *** **alleles and ICH.**

**Figure 4:**
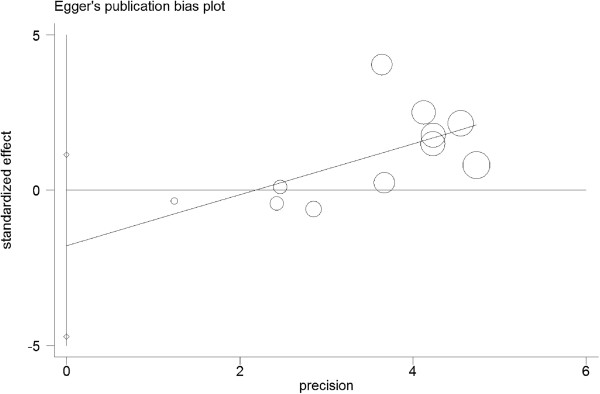
**Egger’s publication bias plot of *APOE *** **alleles and ICH.**

Statistical heterogeneity was not found among studies in overall comparisons by using the Q statistic (Table 3). Publication bias was not found by the Begg's rank correlation method (Figure 3) or Egger weighted regression method (Figure 4).

## Discussion

There is evidence for a role of genetic factors in the development of ICH. Studies investigating the association between genetic polymorphisms and ICH risk are being reported with rapidly increasing frequency. Endoglin gene polymorphism was a risk factor for sporadic ICH [37]. A comparative study that angiotensin converting enzyme (ACE) gene DD homozygosity of the I/D polymorphism in intron 16 is an independent risk factor for ICH in a Polish population [38]. A case–control study suggested that the beta1-tubulin Q43P polymorphism could be associated with ICH in men from southern Spain [39]. A population-based prospective nested case–control study found that estrogen receptor alpha gene polymorphisms were associated with first-ever ICH, particularly in combination with hypertension [40]. A case–control study found that glutathione peroxidase 1 C593T polymorphism was associated with lobar ICH in a Polish population [41]. A case–control study suggested that the rs2228048 of *TGFBR2* gene may be associated with development of ICH in Korean population [42]. A study suggested that the rs17222919 of *ALOX5AP* may be associated with the development of ICH in Korean population [43].

The *APOE* gene polymorphisms are associated with many other diseases. A meta-analysis showed that *APOE* ϵ4 allele appeared to be associated with a higher prevalence of dementia in Parkinson disease [44]. A meta-analysis suggested that the *APOE* ϵ4 isoform was a genetic factor that might influence the age at onset of temporal lobe epilepsy [45]. A meta-analysis showed that the *APOE* ϵ4 allele was associated with an increased risk of developing hypertension [46]. A meta-analysis found that the *APOE* ϵ4 allele was associated with a moderately increased risk for progression from mild cognitive impairment to Alzheimer's disease-type dementia [47]. Prevalence of *APOE* ϵ4 alleles was significantly higher in patients with coronary artery disease than controls [48].

The exact mechanism of the association between *APOE* polymorphism and the risk of ICH remains unclear. *APOE* plays a critical role in redistributing lipids among central nervous system cells for normal lipid homeostasis [49], repairing injured neurons [50], maintaining synaptodendritic connections [51], neurite outgrowth [52], synaptic plasticity [53], mitochondrial resistance to oxidative stress [54], and glucose use by neurons and glial cells [25]. Compared with ϵ3/ϵ3, ϵ4 allele-containing genotypes are associated with increased total cholesterol levels [13]. It appears that the ϵ4 allele enhances amyloid deposition in blood vessels [55]. Thus, one might expect ϵ4 carriers to have increased susceptibility to ICH, especially in a lobar location. Furthermore, *APOE* ϵ4 allele was also associated with an increased risk of developing hypertension [46], which may be the reason that *APOE* ϵ4 allele was associated with a higher risk of ICH.

Several limitations of our meta-analysis should be noted. First of all, meta-analysis is powerful but also controversial-controversial because several conditions are critical to a sound meta-analysis, and small violations of those conditions can lead to misleading results [56]. Second, relatively small sample size of studies in overall comparisons was observed in this meta-analysis. The results of small meta-analyses should be regarded with caution, even if the *P* value shows extreme statistical significance [57]. Thirdly, because of the lack of individual patient data, we could not perform an adjustment estimate. In spite of these limitations, our meta-analysis also had some advantages. First, the major strengths of the meta-analysis are that we used a comprehensive searching strategy based on computer-assisted and manual searching which allowed the eligible studies to be included as far as possible. Second, no heterogeneity or publication bias was found, which leads to a possibly robust result.

In conclusion, our meta-analysis suggested that *APOE* ϵ4 allele was associated with a higher risk of ICH. Future studies will be required to clarify the biological implications of our findings.

## Competing interests

The authors declare that they have no conflict of interests.

## Authors’ contributions

RJZ, XFW, ZCT, JXL, SZY and YBZ carried out the search studies and drafted the manuscript. YJW, WYL and JW, participated in the design of the study and performed the statistical analysis. JLL, BBC and KHZ conceived of the study, and participated in its design and coordination and helped to draft the manuscript. All authors read and approved the final manuscript.
